# Crisis and Human Development

**DOI:** 10.1007/s12124-025-09947-y

**Published:** 2025-12-11

**Authors:** Tania Zittoun, Oliver Clifford Pedersen, Alex Gillespie, Nathalie Muller Mirza, Maeva Perrin

**Affiliations:** 1 University of Neuchâtel, Neuchâtel, Switzerland; 2 London School of Economics and Political Sciences, London, UK; 3 University of Geneva, Geneva, Switzerland

**Keywords:** Crises, Human development, Sociocultural psychology, Dynamic system, Dialogism

## Abstract

Crises are everywhere – from environmental collapses, wars, and skyrocketing inequalities to ageing populations and pandemics, yet there is no universal way that individuals respond to these. What can be said about these? This is an invitation for us, loosely defined as social, cultural and developmental psychologists, to *stop and think*: we emphasise the role crises play in human and cultural development. Our starting point is that, to consider the relevance of crises for human societies and individuals, one needs to address them in time, that is, developmentally; and that one cannot understand development without understanding of crises. Furthermore, a sociocultural psychological perspective is particularly suited for teasing out people’s diverse experiences and perspectives on similar events, and how crises emerge, develop, and resonate in unique ways across life courses. This article first retraces the etymology of the terms *crises* and *developmen*t, and then reviews how crises and development have been articulated in the history of psychology. From there, we turn to the question of crises in development. We present three epistemological principles of our sociocultural stance for studying crises and developments: temporalities and spatialities matters; experiences are dialogical and perspectival; and an idiographic approach offers fertile ground for capturing their complexity.

Crises are everywhere – from environmental collapses, wars, and skyrocketing inequalities to ageing populations and pandemics. Even when we are not personally touched by them, the term “crisis” and its feeling of emergency appear almost daily in the news. Crises can take many forms. They can be global or local, sudden or slow-building, spectacular or invisible, present and on the horizon – and they often bundle together. Naturally, when people live in an environment saturated with crises, they risk falling into a state of paralysis produced by feeling powerless and facing inevitability – as shown in connection with the climate crisis (Badaan et al., 2020). Yet, living through “crises” can also be a call to action, both individually, or collectively – as evident in social movements (Friberg, 2022). There is no universal way that individuals respond to crises (Fassin, 2022), but everyone responds. The response to crisis is idiosyncratic, and it cannot be captured with probabilistic statistics; it is specific, personal, and consequential. So, what can be said about these?

This is an invitation for us, loosely defined as social, cultural and developmental psychologists, to *stop and think* (Arendt, 1978; Valsiner, 2019b). We are not the first to examine crises, but we can offer insight by approaching it from a sociocultural perspective. Our perspective has the specificity of considering both the social and cultural world, and people in it, as well as their mutual transactions (Valsiner, 2000). We emphasise the role crises play in human and cultural development – one may even ask if an organism can grow without crises – and the fact that one needs to consider not only crises as outside observers, but also to give room to people themselves, with their unique perspectives and experiences of these crises.

Our starting point is that, to consider the relevance of crises for human societies and individuals, one needs to address them in time, that is, developmentally; and that one cannot understand development without an understanding of crises. Furthermore, a sociocultural psychological perspective is particularly suited for teasing out people’s diverse experiences and perspectives on similar events, and how crises emerge, develop, and resonate in unique ways across life courses.

This article first retraces the etymology of the terms *crises* and *developmen*t, which ground our dynamic and holistic approach. We then review how crises and development have been articulated in the history of psychology, and distinguish three ways of framing their relation: the crises of psychology; crises as developmental phenomena to be explained by psychology; or societal crises as settings that shape psychological processes. Here, we suggest that the crises of psychology can be overcome by examining the entanglement between societal crises and crises understood as psychological events contributing to human development. From there, we turn to the question of crises in development. We present three epistemological principles of our sociocultural stance for studying crises and developments: temporalities and spatialities matters; experiences are dialogical and perspectival; and an idiographic approach offers fertile ground for capturing their complexity.

##  The Etymological Roots of Crises and Development

The concept of *crisis* entered in English at the beginning of the 15th century (Koselleck & Richter, 2006), and derives from the Greek *krinein*, meaning “to separate, decide, judge” (reconstructed to be from PIE root *krei- “to sieve,” thus “discriminate, distinguish”). It transited through Latin, where *krisis* meant the “turning point in a disease, that change which indicates recovery or death” (used as such by Hippocrates and Galen), literally the “judgment, result of a trial, selection”. Later, it came to mean the “decisive point in the progress of a disease,” also “vitally important or decisive state of things, point at which change must come, for better or worse”; non-medical uses are attested by 1620 in English^1^.

We want to highlight two features of this etymology. First, crisis is anchored in a living system since the term designates a phase in the life of an organism. Second, it appears as vital, marking a point at which change must come, for better or for worse. It thus appears that crisis is a deeply dynamic and temporal term. No wonder, then, that it plays a key role in many developmental approaches, in psychology and beyond.

The etymology of *development* reveals a subtly different set of metaphors. Most dictionaries agree that the term moved from the Latin, through French, and then English in 1650. In old French *desveloper*,* desvoleper*,* desvoloper* means unwrap, unfurl, unveil, or reveal the meaning of, explain; it is built from *des-*, undo and *voloper*, to wrap up^2^ - even though the origin of this “volup” is unclear. According to some, it “is of uncertain origin, possibly Celtic or Germanic”^3^, to others, from the Provençal *viluppo*, envelop. Interestingly, here, development means initially to unwrap from one’s envelop. Here again, we see an organic dynamic and temporal world, except that with development the living entity seems driven by some force which brings it to unfold, unwarp, or extend.

Taken together, the terms crisis and development, or crisis in development, suggest an unfolding and expanding process in which a living entity is engaged, that may come to dramatic moments, where things may become separate, and may turn for the better or for the worse. Thus, crises and development are deeply related. Both are relational terms; something develops into something else, something is undergoing a crisis, or the crisis of something or someone leads to some development. They thus both require a complex and dynamic understanding of organisms understood as a system. But of course, as psychologists or social scientists, what is a crisis, or what or who is affected by a crisis, needs to be further defined.

## Crisis: Starting Conceptions in Psychology and Beyond

The terms development and crises have appeared together in psychology since its inception – to designate the development of the discipline, and of persons. In the 1920 s, Hans Driesch, Karl Bühler and Lev Vygotsky mentioned the crisis of the young field of psychology (Valsiner, 2012). In Vygotsky’s work, it was psychology as a field that was in crisis^4^ in a changing world (Hyman, 2012; Vygotski, 1999). The crisis discourse still regularly comes to the fore in psychology, such as in the 1970 s (Gergen, 1973) and more recently (Lundh, 2019; Pedersen, 2025; Power et al., 2023; Valsiner, 2012).

On a more psychological level, Jean Piaget used the term crisis as a non-theoretical term in an autobiographic essay to designate his own turning point, in a chapter called “la crise” (Piaget, 1918). He also wrote about adolescence and its “adaptation crisis” (Piaget, 1964). Other classical authors have preferred alternative terms to discuss these dynamic moments of catalysed change. For example, *conflict* in Freud’s work (Freud, 1907), *irritation* and *rupture* in Dewey’s (Dewey, 1896), or *conflict* concerning disequilibrium and equilibrium in Piaget’s work (1975).

Erik Erikson extensively used the notion of *crisis* close to its etymological origin. In his life course psychology (Erikson, 1970), the developing person goes through a series of crises, each of them producing either a form of resolution/integration, or fragmentation (Erikson, 1968). It is also interesting to note that, for him, the development of a person could not be understood outside the cultural and historical context, itself going through its own development (Erikson, 1958, 1969). This paved the way for a developmental psychology examining interactions between developing individuals and their ever-changing world.

In neighbouring social sciences, the use and exploration of “crisis” have proliferated massively, and recently scholars have questioned definitions that lean too heavily on the concept’s etymological roots – a clearly demarcated event that sits between two states of normality (Fassin, 2022; Vigh, 2008). For example, Mbembe and Roitman (1995) have argued that in some post-colonial African contexts, crisis is not a temporary state of exception but part of the ordinary condition. They therefore invite scholars to dwell on how people navigate, develop, imagine and create lives under these crisis conditions – in other words, how they become the “crisis figure”. Rather than assuming the world is inherently benign, and approaching crises as hurdles along the linear flow of time, these studies recognise that crisis is an enduring state for many people worldwide (Scheper-Hughes, 2008). Vigh’s (2022) research in Bissau on chronic or slow-crises epitomises this point, and he proposes that the crises should be viewed as the context of inquiry, not “single aberrations” to be understood.

Other social scientists have shown how crises can become displaced in time and space to exert a slow, often invisible violence over time (Ahmann, 2024; Nixon, 2011), or how labelling something as a crisis is an inherently political act (Roitman, 2014), which exerts power over what it is and how urgent it is. For example, Indigenous scholars have shown the discrepancies between the hegemonic discourse portraying the climate crisis as being on the horizon and their experiences of already having lived through it (Cunsolo et al., 2020; Whyte, 2017). Beyond the obvious interest of these approaches, they however raise the question of perspectives: who defines what constitutes a crisis, researchers or participants? And what does it mean, for whom?

We acknowledge that crises can take different forms, and can occur with different temporalities, intensities, and scales (Bergman-Rosamond et al., 2022). We also accept that crises are not static, but dynamic events that may change composition through time and in response to events (Zhukova, 2022). Lastly, we also want to highlight how people experience crises and how they may become psychologically significant. Accordingly, it is important to distinguish who speaks of crises, from which perspective, to whom, and for what purpose. Only then can we explore the gap between the very strict Académie française, which prevents to use “crisis” too widely (as “it should be reserved for precise phenomena and events limited in time”^5^), and the many ways various persons experience them.

## Three Levels of Crisis in Developmental Psychology

Examining “crisis” together with “development” invites us to distinguish three problems.

First, *developmental psychology* is among the many subdomains in psychology touched by “the crisis” of the discipline. Developmental psychology started as a developmental science – a science of unfolding – from Piaget’s careful observations of children’s growth to Vygotsky’s observations of children and adults in his close and professional environments (Perret-Clermont & Barrelet, 2008; Piaget & Romy, 1912; Zavershneva & Van der Veer, 2018). However, in the search for a more scientific and systematic science, developmental psychology increasingly focused on phases and cross-comparisons more than longitudinal processes (Säljö, 2022). The majority of studies in developmental psychology present no actual process: states are compared (pretest and post-test, different age groups, etc.). There is, however, a small and consistent number of researchers who emphasize processes in developmental psychology.

In their 2003 *Handbook*, Jaan Valsiner and Kevin J. Connolly posed general principles for psychology as a developmental science:

For development to occur, irreversible changes in organisation are necessary; this in turn required an open system. An open system is one having exchange relationships with its environment. The environment of the developing organism is its context. The idea of context is relative to the developing organism. It does not exist independently of that organism. Hence any investigator who studies development must necessarily take into account the context of the developing system (Valsiner & Connolly, 2005, p. x).

In addition, development is characterised by activity: the organism grows and changes in complexity to adjust to the demands of a changing environment, at various levels of complexity, sometimes becoming more complex, sometimes less. Eventually, autonomous activity may allow the organism to anticipatory changes and develop self-reflexivity. Also, organisms develop within multiple, non-linear systems - that is, through emergence, often via catalysis (see also Valsiner, 2019a). Fundamentally, developmental science is not the study of what *is*, but of what is *becoming*. Thus, the authors conclude their chapter with a quote of Alan Turing: “most of an organism, most of the time, is developing from one pattern to another, not from homogeneity into a pattern” (Valsiner & Connolly, 2005, p. xvii). Hence, there is still a crisis in developmental psychology, but there are principles that may guide us towards learning and the development of the field; taking into account temporality, complexity, context, activity, and processes of creative emergence are key for a developmental psychology beyond its own crisis.

Second, crises can be considered as part of the process of human development. Indeed, studying changing adjustments between the organism and its environment requires identifying moments of change. The notion of crises, and its related semantic field – rupture, conflict, etc. (see also Gillespie & Zittoun, 2025) – is one of the good candidates to do so. In this *theoretical meaning* of the term, crisis may indeed arise from a mismatch within the organism – at one or diverse levels of organisation – or between the organism and its environment, including any of its subparts – relations with other organisms, persons, non-humans, or the demands of the material or symbolic world. This may entail mismatches between collective meaning and personal sense-making, meaning and action, people’s embodied and affective experience, and what the environment affords them. It may also be a mismatch between different goals people pursue, or different subparts of their experience or identities (Chahraoui, 2014; Erikson, 1968; Lima & Hviid, 2022). From a theoretical perspective, thus, crisis is a key element in development itself. These processes triggered by crises are loosely characterised by various authors: equilibration in Piaget, higher-order semiotic mediation in the Vygotskian tradition, dialogical resolution in other fields. Thus, to progress in theorising development, one needs a more subtle and differentiated understanding of the psychological response to a crisis.

Third, insofar as developmental science studies the changing interactions between the organism and the environment, it is unfortunately the case that our current environment is in trouble. Among the many changes that we must adjust to are the unexpected, disturbing events that affect many people and/or the environment at once. These are *societal* crises – crises affecting large groups of the population in the world, and that common sense usually identifies as crises. This is where the COVID-19 pandemic, wars affecting the lives of millions of people, radical polarisation in local and international politics, and environmental destruction, all appear as “crises”. These, of course, affect the developmental trajectories of people, and therefore psychological studies are increasingly examining the developmental consequences of these crises (e.g., Alhadeff-Jones, 2021; Asiamah et al., 2023; Benasayag & Schmit, 2003; Lass-Hennemann et al., 2024; Pedersen, 2025; Rajala et al., 2023; Stenner, 2022; Zittoun, 2023).

In the present special issue, we address these three aspects of crises: the crisis of developmental psychology by taking seriously crisis and change; we try to better understand the core process of change by analysing it through detailed cases; and we theorise the developmental dynamics that take place when people experience societal crises. Hence, perhaps the solution to the crises of psychology is to study complex wholes, in which historically and culturally situated phenomena engage both societal crises and their more personal counterpart, and to understand how both ranges of phenomena partake to human and social development. In that sense, we examine crises in/and development.

## A Sociocultural Integrative View of Crises in/and Development

As suggested, understanding crises and development requires treating them as dynamically related in and across people’s lives. An open dynamic systems’ approach provides the metatheoretical frame for this view: crises emerge within complex systems of different unities as disruptions of dynamic equilibrium. These give rise to processes of new equilibria – around new attractors, through reorganisation toward greater or lesser complexity, or sometimes through the creation of new entities (van Geert, 2003, 2019; Witherington, 2007).

That said, the type of phenomenon we approach as social, cultural and developmental psychologists requires further elaboration. In effect, our specificity is that we consider the mutual constitution of developing persons and their evolving sociocultural and material environments (Muller Mirza & Dos Santos 2019; Pedersen, 2022; Valsiner, 2000; Zittoun & Gillespie, 2015). This necessitates that we examine phenomena of different nature, over various temporalities, either alternatively or together. Equally, crisis can have various scales, in terms of their geographical expansion, the number of people affected, and the duration of the crisis and its resolution. Let us imagine the concrete case of an average adolescent in central Europe today: they may simultaneously be experiencing the crisis of their growth into young adults, the crisis of their first romantic breakup, an economic crisis in their vocational education, a political crisis through their daily discussions with young refugees, and a climate crisis through waves of summer canicule. We could take any other example of a person’s life in society: the complexity of simultaneous, parallel, interrelated crises would be equivalent. How do these various personal and societal crises interact? How can people develop through these dynamics? How can they act upon them? How can we, sociocultural psychologists, define a theoretical frame able to capture these complexities of crises and/in development?

A sociocultural approach to crises and development starts by acknowledging the inherent complexity of the phenomena. We propose three epistemological principles for the study of crises in/and development, two theoretical and one more methodological: (1) the multiplicity of spatial and temporal scales; (2) the dialogical and perspectival nature of crises; (3) the necessity for a case-study, or ideographic approach, to investigate crises and/in development. Let us examine each principle in turn.

1. The multiple spatial and temporalities scales of crises in/and development.

To approach the complexity of developmental dynamics in situated lives, sociocultural psychology often draws upon a distinction that has been worked out over decades, growing from Vygotsky’s work, among others (Van der Veer & Valsiner, 1993; Zittoun, 2025). It distinguishes sociogenetic processes, from microgenetic, and ontogenetic ones. Of course, other authors prefer distinctions between macro and microprocesses à la Bronfenbrenner (1979), or comparable levels of analysis (Doise, 1980; Perret-Clermont, 2015): yet the reasoning is comparable.

Sociogenesis designates transformations of the social world, that is, the political, institutional, material, natural, and symbolic world in which people live. Sociogenetic processes include political upheavals, revolutions, changes in policies, urbanisation, the transformation of social representations, etc. Typically, what common sense designates as “crisis” are societal crises, that is, crises affecting sociogenesis. Of course, one may always ask from whose perspective a “crisis” is defined.

Microgenesis designates changes occurring in the here-and-now of a given situation. It is about interactions between people, between people and their environment, or even between inner voices or positions. Crises at this level may emerge as a dispute, a conflict, a resistance from the environment. Societal crises manifest in everyday experiences; a country at war is felt in the struggle to secure daily food; or a pandemic is experienced in the microgenetic act of disinfecting one’s hands.

Ontogenesis refers to individual development across the life course. Crises can occur within one’s development due to changes in the environment or one’s physical and psychological needs and wants. For example, the crisis around retirement usually occurs when a person is required to stop their professional activity and are treated as “senior” from one day to the other, when they still feel engaged and striving to contribute to society. However, human ontogenetic development in a country at war, spending one’s students’ years locked in a flat, are also occurrences of societal crises shaping ontogenetic dynamics.

Hence, from these embedded genesis, one can distinguish two mutually dependent dimensions: scale and temporality. Of course, it may be artificial to separate time from space; after all, we are embedded in various time-space configurations, as the notion of chronotope suggests (Bakhtin, 1996a; Marková & Novaes, 2020). Yet for analytical purposes, let us distinguish them. In effect, these can vary both in terms of what is considered analytically and what is relevant empirically. To speak of socio, micro and onto genesis is to designate the change at the level of the social, interactions, or singular persons. Intuitively, one would say that the social is wider (in spatial extension) and slower, while microgenesis is smaller and quicker. However, things are far more complex.

Consider sudden catastrophes at the sociogenetic level: the collapse of the World Trade towers in 2001 took a couple of hours and was local, yet it changed the course of world history and still has consequences today. The Bhopal gas leakage in the 1980 was also relatively rapid and local, and although it shocked world opinion, it remained relatively local in its consequences (Gillespie & Zittoun, 2025; Stenner, 2025). A the level of microgenesis, a couple may suddenly collapse because of a wrong gaze or sentence, as in the French film *le Mépris* (Godard, 1963), or because of the slow misalignment of two persons’ attention over years (Bergman, 1975) (Neuman, 2025). At a more ontogenetic level, a person’s professional crisis may have an effect over months or even years, but a crisis related to parenting choices may have life-long consequences (Muller Mirza, 2025). Also, one may think that obtaining citizenship affects mainly one person – an ontogenetic phenomenon, with limited temporal and spatial extension. But in cases of asylum-seekers in the UK, the journey can cross continents (Crafter et al., 2025; Power & Botelho, 2025); and the procedure may take years (Di Donato et al., 2020).

Yet it is still more complex: the spatial and temporal dimensions can be combined in various ways. On a grand scale, the climate crisis has been slow-burning for decades, yet some places are already feeling its ramifications; it may be progressively experienced by a singular person as a crisis over years (Pedersen & Perrin, 2025); and it may require a very fast worldwide response. The COVID 19 pandemic was worldwide, collective, and short-lived, yet at the scale of a person, it may be a life-changing experience (Wagoner & Herbig, 2025). And again, societies and people rarely experience just “one crisis”. Usually, over a shorter or longer time span, crises are likely to be experienced interdependently, more or less resonating crises, such as when the AIDS crisis becomes linked and compared with the COVID-19 pandemic (Pedersen et al., 2025) or when one’s teenage crises are re-experienced within a retirement crisis (Zittoun & Gillespie, 2025).

This first epistemological principle thus implies that under the umbrella term “crisis”, used to designate complex social transformations, processes of varying complexity, amplitude and duration, there are usually multiple interacting crises simultaneously. It also implies that the same event, observed from the outside, may be experienced very differently by different persons, in different positions – or even by the same persons at different moments.

2. The dialogical and perspectival nature of crises in/and development.

To disentangle the temporal and spatial complexities of crises, one may begin by distinguishing perspectives from which these are experienced. In effect, people are unique, and occupy different positions in the social, material and symbolic world, from which they develop their perspectives upon others and events (Gillespie, 2021; Gillespie & Martin, 2014). Perspectives matter because these are the standpoints from which a crisis can be identified, silenced or named, and communicated about and acted upon or not. Perspectives may be that of the researchers versus that of research participants, politicians and institutions representatives versus single persons, persons in (institutional, political, financial) power over dependent persons on a given matter (teachers versus students, civil servant versus asylum seeker, etc.), or even can take place within a person, when experiencing ambivalence, or when reconsidering one’s past (Muller Mirza, 2025, Zittoun & Gillespie, 2025).

The right to identify, name, hide or act upon a crisis may have consequences for other people, in other positions, with different perspectives. In any case, these have consequences, in allowing, supporting, catalysing or impeding change and development. Two married adults may have very different perspectives on each other and the nature of the crisis in their marriage (Neuman, 2025). During a negotiation of citizenship rights, the desk officers have to identify how plausible the narratives of child asylum seeker are, and are engaged in identifying inconsistencies, so as to apply guidelines in vigour, while asylum seekers have to convince these officers that they are vulnerable, regardless of the skills and resources they used to cross half of the world (Crafter et al., 2025; Power & Botelho, 2025). The lack of consideration and communication by the US government about the AIDS pandemic in the 1980s affected the lives of hundreds of thousands of people, who alone, and then in locally organised groups, responded to that crisis (Pedersen et al., 2025; Zittoun & Gillespie, 2025). On the other hand, one person may believe that she is lied to by the authorities about the COVID 19 pandemic, and be searching for alternative perspectives (Wagoner & Herbig, 2025).

Not only are crises in/and development perspectival, but these are also dialogical. A crisis is never the crisis of one person, nor is it a crisis of one moment. Once we adopt a temporal, developmental view, it is clear that a crisis has always antecedents and consequences (Gammeltoft-Hansen et al., 2022). It is also always echoing and reflecting other phenomena and generating new ones. Incidentally, a crisis affecting one environment, a group or a person, is always also consequential for other environments, groups or persons. In other words, crises are best apprehended from a relational, or more specifically, dialogical perspective (Bakhtin, 1996b; Marková, 2016, 2023). In effect, when one considers even the most minute encounter, these dialogical dynamics immediately come to the fore.

Consider the case, in the “migration crisis”, of an encounter between a professional who has to decide whether a young person classified as an “unaccompanied minor” has the right to stay in the country in which they seek asylum (Crafter et al., 2025). Of course, this will occur in dialogue between persons. This may be underpinned by the professional’s inner dialogue, between a wish to take care of a young person and administrative guidelines. This microgenetic encounter reflects and contributes to sociogenetic transformation (e.g., geopolitics, the harshening of asylum rights), and it will radically impact the ontogenesis of that young person. What occurs in the microgenetic here and now is also part of chains with histories (the refugee’s lifecourse, their country’s history, the caseworker’s past experiences, and the societal debate about immigration) and these, in turn, shape futures (the refugee becomes a citizen, the caseworker is promoted, society becomes more or less diverse). Taking these aspects seriously reveals that a person’s “past” includes not only various crises, but also elements that may become resources in the future. The past of the professional – through her experience, sensitivity, personal circumstances – may affect her consideration of the child and their future. Policy, in turn, is related to political and power games, strategic goals, economic variations, and other elements with their own past and future, which constrain the field of possibilities within the here and now.

Interestingly, if crises can be apprehended differently depending on people’s perspectives within complex dialogical dynamics, it is also often (but not always) these different perspectives and dialogical positions that trigger crises, and that are occasions for development. A marital crisis may lead one of the spouses to open new developmental paths (Neuman, 2025); a major political crises may lead a youngster to open new life options abroad (Crafter et al., 2025; Power & Botelho, 2025); a sanitary or industrial crises due to a lack of listening between different perspectives may lead organisations to implement better security procedures (Gillespie & Zittoun, 2025, Stenner, 2025).

In other words, any event linked to a crisis can be apprehended from multiple perspectives; it has multiple determinations and multiple consequences. Not only does it have multiple causalities, as suggested by a developmental perspective, but it also opens an infinity of possibilities. It has resonances at various levels of determination and different temporalities (Pedersen et al., 2025). “A” crisis is thus often only a part of a complex phenomenon, where many more determinations occur which are equally treatable as “crisis”. Within a crisis, there are possibilities to change the future, to avert a worsening crisis, to change one’s life, or to prevent the next crisis. In this sense, crises often have a world-making element (Power et al., 2023).

Our sociocultural approach thus suggests that no event or crisis in development is an isolated event. All crises are already perspectival, dialogical, and imply multiple developmental lines. What is affected are always complex relations – between people, and between people and their material, social and symbolic environment.

3. The necessity of approaching crises in/and development through case-studies.

The third epistemological principle for a sociocultural approach to crises and/in development is methodological, and it follows from the two previous ones. In order to capture the complexity of multiscale, spatial and temporal phenomena, from a perspectival and dialogical perspective, then phenomena need to be approached using a subtle case-study approach.

In effect, one must identify a phenomenon (a person’s life, a type of interaction, a relationship, a human failure) and then capture its dynamic while documenting its environment in time and space. Indeed, one cannot predefine that this real-life phenomenon constitutes a crisis of this or that, or that development will follow a specific path. Defining a historically and socio-culturally embedded phenomenon necessitates patiently analysing them in their particularity over time.

Complex case studies allow us to apprehend phenomena at various interrelated scales and temporalities, and their mutually evolving dynamics (Flyvbjerg, 2011). Complex case studies undertaken from a dialogical stance have a certain number of additional characteristics. They emphasise the active role of persons:

Dialogical case studies prioritise the role of human agency in this complexity, insisting that a case is not composed of multiple physical elements, but composed by humans actively making sense of embodied, interested, socially positioned, agentic self-other dialogues (Cornish, 2020, p. 142).

In addition, these cases are dialogical; they foreground the multiple relationality and temporality of the dynamic at stake. Eventually, a dialogical account has an ethical implication: it implies acknowledging the unique otherness of the others involved in a study, and the relationship one has, as a researcher, toward them (Cornish, 2020; Marková et al., 2020).

In this special issue, we thus invited contributors who document complex realities, through case studies at different scales and temporalities to theorise crises and development. Most contributors approached them from an inherently dialogical perspective. Whatever developmental dynamics are foregrounded, they are examined in the context of other crises and developmental dynamics.

## The Special Issue

Based on these three epistemological assumptions, all the papers in this special issue contribute to our understanding of crises and/in development.

Paul Stenner (2025) proposes a process ontology to question the nature of liminal dynamics engaged in crises and/in development. Alex Gillespie and Tania Zittoun (2025) question whether crises are really a core component of development, or whether researchers are not blinded by overarching cultural narratives of crises obscuring our theoretical imagination.

The other articles are all based on case studies, approaching the question of crises and/in development at diverse scales. Some of the case studies open a window into microgenetic processes, for example, the interactions between caseworkers and people requesting asylum, showing the dialogical or power dynamics at sake (Crafter et al., 2025; Power & Botelho, 2025). As suggested, these interactions can only be understood in the context of complex and intermeshed systemic crises, such as the socio-political crises (Power & Botelho, 2025), the migration crisis (Crafter et al., 2025) and the climate crisis (Perrin & Pedersen, 2025), shaping the here-and-now of an interaction and the developmental trajectory of a young person. Other articles focus on interactions across a longer time span. For example, Neuman (2025) uses vectors to analyse dialogues within a couple in marital crises over a couple of years, exploring their relational crisis. Others (Gillespie & Zittoun, 2025; Stenner, 2025) examine institutional interactions that lead to an industrial crisis over months due to denial of warning signs. Some articles foreground ontogenetic dynamics by constructing case studies of single persons over time, examining a couple of years in the life of a person marked by one major crisis or many potential crises, showing the role of analogical reasoning and sense-making (Wagoner & Herbig, 2025; Muller Mirza 2025). Others have followed people through decades of diary writing, detailing how the climate crisis evolves for a person and their imaginations (Perrin & Pedersen, 2025), which eventually contribute to complex developmental dynamics in the life of adults (Zittoun & Gillespie, 2025). These case studies support the idea that societal crises do not always produce personal ruptures. Also, complex case studies reveal that crises are contested, imagined, real, fractured, overlapping, multiple, and more.

Put together, these case studies contribute to developmental science through a focus on the dynamic particularities of individual cases, without ever reducing them to “average cases”. They point toward new conceptual tools to analyse crises (Gillespie & Zittoun, 2025; Stenner, 2025) and developmental dynamics, both as essential phenomena (Valsiner, this issue) or from a life course perspective (Muller Mirza, 2025; Zittoun & Gillespie, 2025).

Taken together, these articles approach crises and development by combining various levels of analysis and temporal scales. The case studies demonstrate the need for complex single case studies to study human development through crises (Cornish, 2020; Marková et al., 2020). These rich case studies are needed to maintain the thick descriptions within developmental approaches, and to firmly theorise the processes at stake. Such cases are the ground-truth that theorisation of crises in/and development must start with. Thus, we propose, the careful consideration of crises in/and development from a sociocultural psychology perspective may advance the longstanding crisis of psychology: returning it to the dynamic particulars of what actually goes on.

## Data Availability

No datasets were generated or analysed during the current study.

## Footnotes

1
https://www.etymonline.com/word/crisis.

2
https://www.etymonline.com/word/development.

3
https://www.etymonline.com/word/development.

4 He used the Russian term “krizisa”.

5 « Le mot *Crise* est souvent employé abusivement. Il devrait être réservé à des phénomènes précis et à des évènements limités dans le temps ». https://www.dictionnaire-academie.fr/article/A9C4991.
